# Supplementary material for: Investigating post-COVID-19 confidence in emergency use authorization vaccines: A hypothetical case of mpox
Source: PLoS Negl Trop Dis. 2025 May 29;19(5):e0013037. doi: 10.1371/journal.pntd.0013037 (PMC12187215; doi:10.1371/journal.pntd.0013037)
Supplement: S1 Appendix — (DOCX) [file pntd.0013037.s001.docx]

**Vaccine Confidence Post-COVID-19 on Future Pandemics:**

**A Hypothetical Case of Mpox Vaccine**

**Section A: General information**

| 1 | Age | __________ years old |
| --- | --- | --- |
| 2 | Gender | [ 1 ] Male  [ 2 ] Female |
| 3 | Highest educational level | [ 1 ] Primary school and below  [ 2 ] Secondary school  [ 3 ] High school/technical school  [ 4 ] Strongly disagree  [ 5 ] Bachelor  [ 6 ] Masters/PhD |
| 4 | Family annual income (CNY) | [ 1 ] <50,000  [ 2 ] 50,000 – 100,000  [ 3 ] 100,001 – 150,000  [ 4 ] 150,001 – 200,000  [ 5 ] 250,001 – 300,000  [ 6 ] >300,000 |
| 5 | Locality | [ 1 ] Urban  [ 2 ] Rural |
| 6 | Region | [ 1 ] Northern  [ 2 ] Northeastern  [ 3 ] Eastern  [ 4 ] Southern Central  [ 5 ] Southwestern  [ 6 ] Northwestern |

**Section B: Knowledge about Mpox symptoms**

| 1 | Rashes (that may be located on hands, feet, chest, face, or mouth or near the genitals, including penis, testicles, labia, and vagina, and anus) | [ 1 ] True [ 2 ] False [ 3 ] Don’t know |
| --- | --- | --- |
| 2 | Fever | [ 1 ] True [ 2 ] False [ 3 ] Don’t know |
| 3 | Chills | [ 1 ] True [ 2 ] False [ 3 ] Don’t know |
| 4 | Swollen lymph nodes | [ 1 ] True [ 2 ] False [ 3 ] Don’t know |
| 5 | Exhaustion | [ 1 ] True [ 2 ] False [ 3 ] Don’t know |
| 6 | Muscle aches and backache | [ 1 ] True [ 2 ] False [ 3 ] Don’t know |
| 7 | Headache | [ 1 ] True [ 2 ] False [ 3 ] Don’t know |
| 8 | Respiratory symptoms (e.g., sore throat, nasal congestion, or cough) | [ 1 ] True [ 2 ] False [ 3 ] Don’t know |

**Section C: Perception towards Mpox**

| 1 | **Perceived Susceptibility**  How likely do you believe it is that you know will contract Mpox in the near future? | [ 1 ] Not at all likely  [ 2 ] Not very likely  [ 3 ] Somewhat likely  [ 4 ] Very likely |
| --- | --- | --- |
| 2 | **Perceived Severity**  How serious do you think Mpox would be if you were to contract it? | [ 1 ] Not severe at all  [ 2 ] Slightly severe  [ 3 ] Moderately severe  [ 4 ] Very severe |
| 3 | **Fear of Mpox**  How concerned are you about the potential risks and consequences of Mpox? | [ 1 ] Not concerned at all  [ 2 ] Slightly concerned  [ 3 ] Somewhat concerned  [ 4 ] Very concerned |

**Section D: Practice of recommended precautions to prevent Mpox infection**

| As of now, what preventive measures are you currently taking to avoid Mpox? | | |
| --- | --- | --- |
| 1 | **Avoid Close Contact**  Avoid close physical contact with people who have a rash or are suspected to be infected with Mpox. | [ 0 ] Never  [ 1 ] Sometimes  [ 2 ] Often  [ 3 ] Always |
| 2 | **Practice Good Hygiene:**  Wash your hands frequently with soap and water, or use an alcohol-based hand sanitizer, especially after contact with potentially infected individuals or animals | [ 0 ] Never  [ 1 ] Sometimes  [ 2 ] Often  [ 3 ] Always |
| 3 | Avoid touching your face, particularly your eyes, nose, and mouth, with unwashed hands. | [ 0 ] Never  [ 1 ] Sometimes  [ 2 ] Often  [ 3 ] Always |
| 4 | **Use Personal Protective Equipment (PPE):**  Wear a mask in crowded places | [ 0 ] Never  [ 1 ] Sometimes  [ 2 ] Often  [ 3 ] Always |
| 5 | **Stay Informed**  Keep updated with public health guidelines and advice from health authorities regarding Mpox | [ 0 ] Never  [ 1 ] Sometimes  [ 2 ] Often  [ 3 ] Always |

**Section E: Attitudes towards the use of an Emergency Use Authorization (EUA) vaccine to curb a pandemic of an infectious disease**

| 1 | I am concerned that an EUA vaccine may have been approved too quickly without adequate testing. | [ 1 ] Strongly agree  [ 2 ] Agree  [ 3 ] Disagree  [ 4 ] Strongly Disagree |
| --- | --- | --- |
| 2 | I have concerns about the quality control and manufacturing processes of an EUA vaccine. | [ 1 ] Strongly agree  [ 2 ] Agree  [ 3 ] Disagree  [ 4 ] Strongly Disagree |
| 3 | I am concerned that an EUA vaccine may not be as effective as a fully approved vaccine. | [ 1 ] Strongly agree  [ 2 ] Agree  [ 3 ] Disagree  [ 4 ] Strongly Disagree |
| 4 | I am skeptical about the transparency of the approval process for an EUA vaccine. | [ 1 ] Strongly agree  [ 2 ] Agree  [ 3 ] Disagree  [ 4 ] Strongly Disagree |
| 5 | I doubt the effectiveness of an EUA vaccine compared to vaccines that have undergone full regulatory approval. | [ 1 ] Strongly agree  [ 2 ] Agree  [ 3 ] Disagree  [ 4 ] Strongly Disagree |
| 6 | I worry that there could be long-term side effects from an EUA vaccine that are not yet known. | [ 1 ] Strongly agree  [ 2 ] Agree  [ 3 ] Disagree  [ 4 ] Strongly Disagree |
| 7 | I believe that an EUA vaccine is a necessary tool for controlling the spread of infectious diseases during a pandemic. | [ 1 ] Strongly agree  [ 2 ] Agree  [ 3 ] Disagree  [ 4 ] Strongly Disagree |
| 8 | I believe that the benefits of using an EUA vaccine outweigh any potential risks associated with its use during a pandemic. | [ 1 ] Strongly agree  [ 2 ] Agree  [ 3 ] Disagree  [ 4 ] Strongly Disagree |
| 9 | I would be willing to receive an EUA vaccine if it is the only option. | [ 1 ] Strongly agree  [ 2 ] Agree  [ 3 ] Disagree  [ 4 ] Strongly Disagree |
| 10 | The use of an EUA vaccine might undermine public trust in vaccines overall. | [ 1 ] Strongly agree  [ 2 ] Agree  [ 3 ] Disagree  [ 4 ] Strongly Disagree |

**Section F: Lessons Learned from Emergency Use Authorization (EUA) COVID-19 Vaccination on Confidence in a Future EUV vaccine for Mpox**

| 1 | To what extend the EUA COVID-19 vaccination affected your trust in future EUA vaccine for pandemic of infectious disease such as Mpox? | [ 1 ] My trust has significantly decreased  [ 2 ] My trust has somewhat decreased  [ 3 ] My trust has remained the same  [ 4 ] My trust has somewhat increased  [ 5 ] My trust has significantly increased |
| --- | --- | --- |

**Section F: Willingness to be vaccinated against Emergency Use Authorizations (EUAs) vaccine for Mpox**

| 1 | If a vaccine for Mpox was developed and ***authorized for emergency use***, would you be willing to receive it? | [ 1 ] Extremely Willing  [ 2 ] Somewhat willing  [ 3 ] Undecided  [ 4 ] Somewhat not willing  [ 5 ] Not willing |
| --- | --- | --- |
| 2 | If a vaccine for Mpox was developed and ***authorized for emergency use***, would you be willing to receive an ***mRNA vaccine, a traditional vaccine, or either type*** if available? | [ 1 ] mRNA only  [ 2 ] Traditional conventional vaccine only  [ 3 ] Either type of vaccine  [ 4 ] I do not want to receive any Mpox vaccine  [ 5 ] I’m not sure about the difference between mRNA and traditional vaccines.  NOTE:  An **mRNA vaccine** is a type of vaccine that uses messenger RNA to instruct cells in the body to produce a protein that triggers an immune response. This response helps the body recognize and fight the actual virus if it encounters it in the future.  On the other hand, a **conventional (traditional) vaccine** typically uses a weakened or inactivated form of the virus, or a protein from the virus, to stimulate the immune system to recognize and combat the virus without causing the disease. |

**Section H: Willingness to pay for Mpox vaccine**

| 1 | How much would you be **willing to pay** for a vaccine that protects against Mpox if it becomes available? | [ 1 ] I am not willing to pay for the Mpox vaccine  [ 2 ] I would only get the vaccine if it were free  [ 3 ] I am willing to pay any amount necessary  [ 4 ] I am willing to pay a certain amount  If answer [4], proceed to the next question, where we would like to know the maximum price that you will pay for a dose of Mpox vaccine. |
| --- | --- | --- |
| 2 | How much is the maximum price that you will pay for a dose of Mpox vaccine if available? | CYN (YUAN) _____________ |
