# Supplementary figures and images for: Investigating post-COVID-19 confidence in emergency use authorization vaccines: A hypothetical case of mpox
Source: PLoS Negl Trop Dis. 2025 May 29;19(5):e0013037. doi: 10.1371/journal.pntd.0013037 (PMC12187215; doi:10.1371/journal.pntd.0013037)

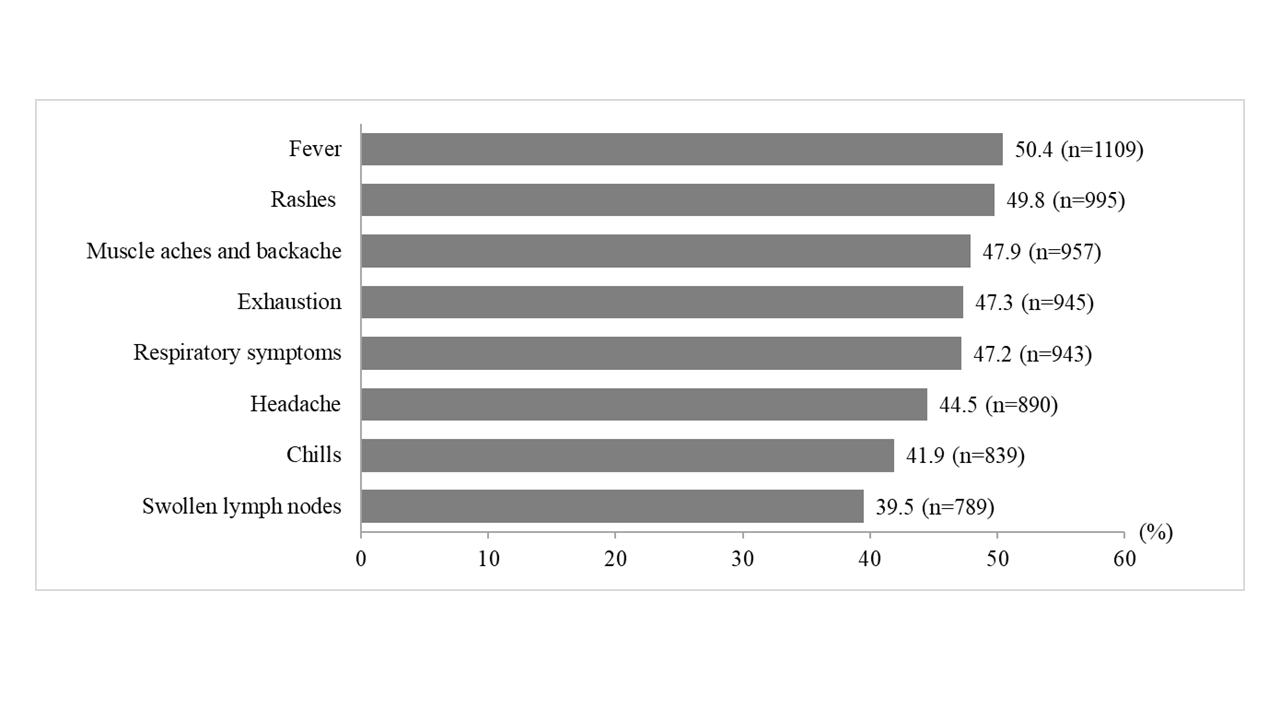

Supplement: S2 Appendix — (TIF) [file pntd.0013037.s002.tif]

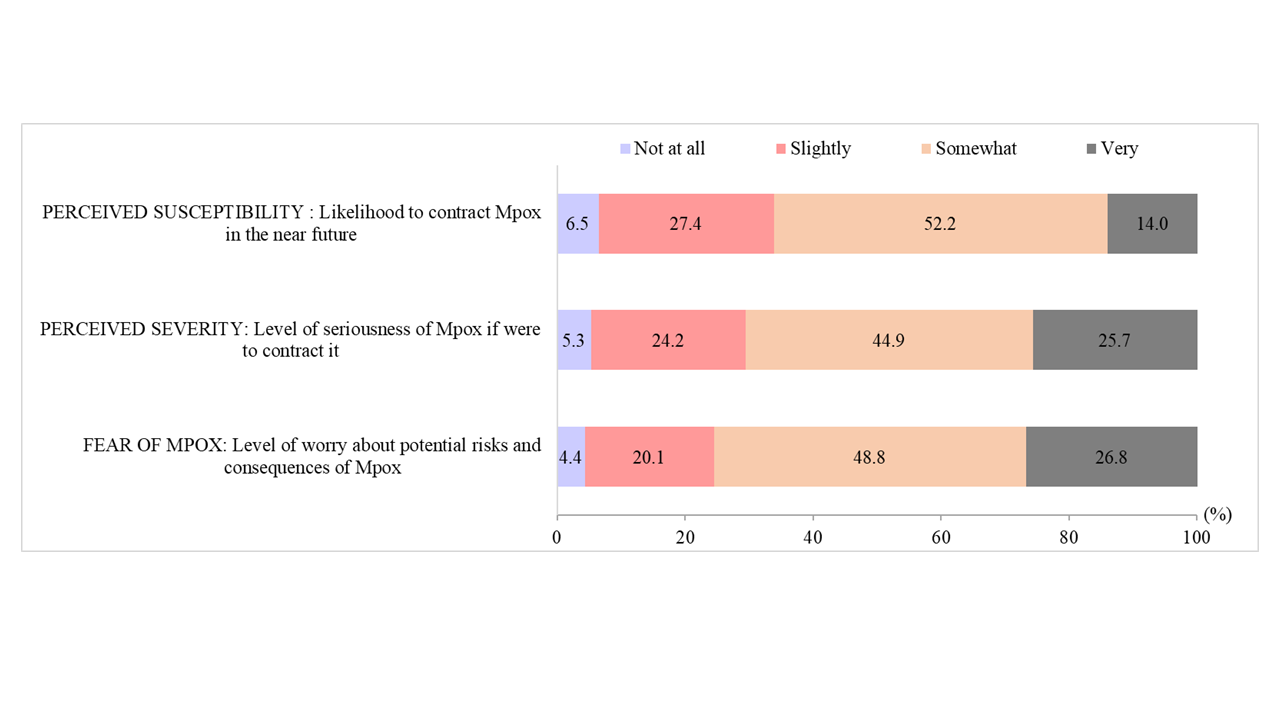

Supplement: S3 Appendix — (TIF) [file pntd.0013037.s003.tif]

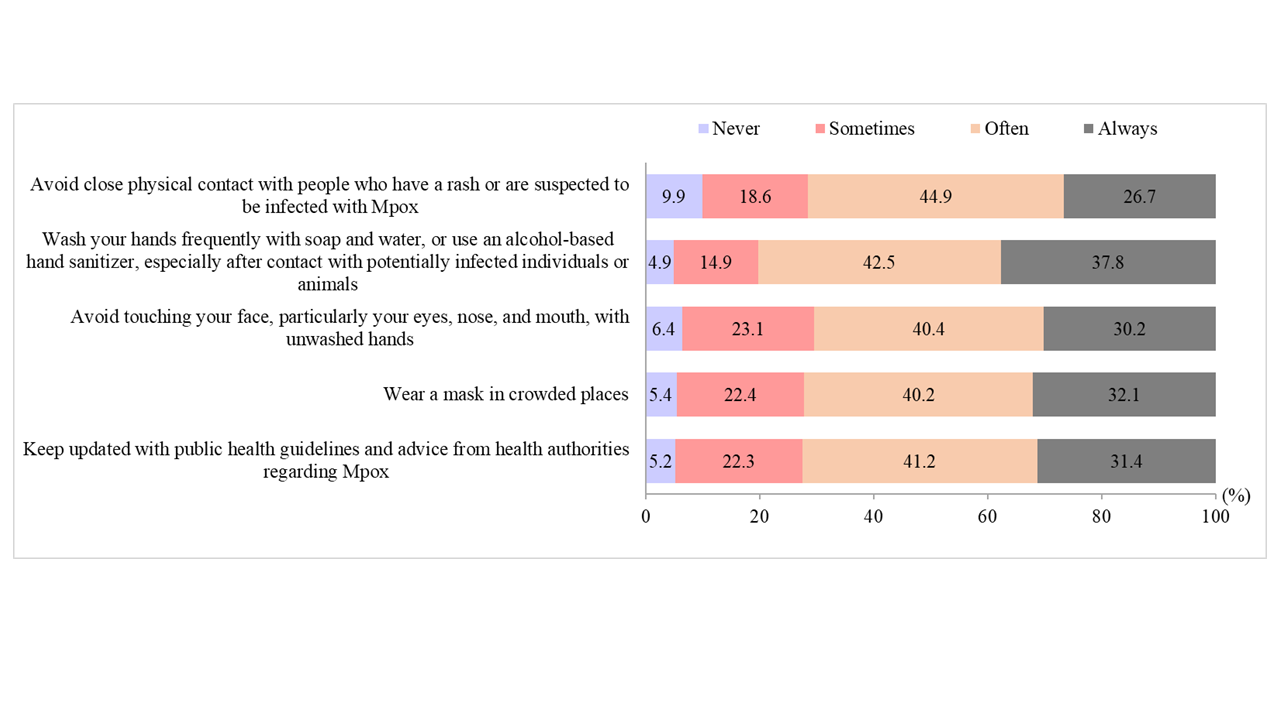

Supplement: S4 Appendix — (TIF) [file pntd.0013037.s004.tif]

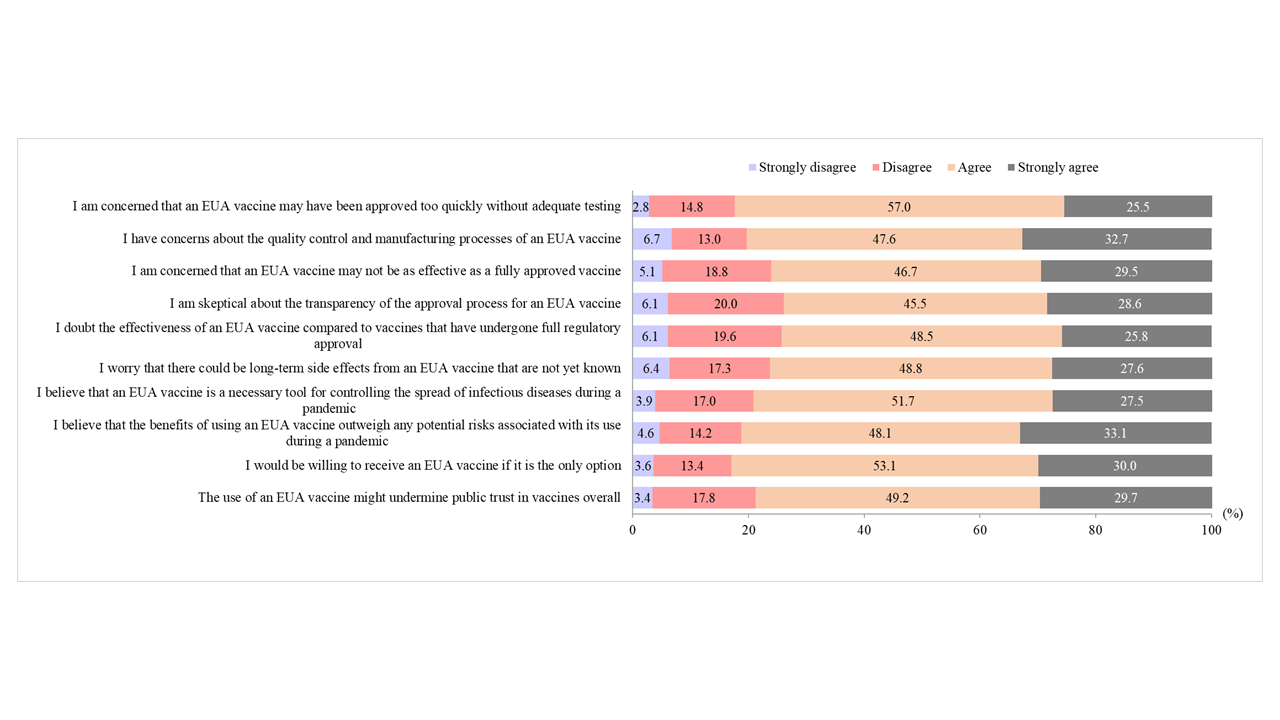

Supplement: S5 Appendix — (TIF) [file pntd.0013037.s005.tif]
