# Supplementary material for: Investigating post-COVID-19 confidence in emergency use authorization vaccines: A hypothetical case of mpox
Source: PLoS Negl Trop Dis. 2025 May 29;19(5):e0013037. doi: 10.1371/journal.pntd.0013037 (PMC12187215; doi:10.1371/journal.pntd.0013037)
Supplement: Attachment — Submitted filename: Respond to reviewer 1.03.2024.doc [file pntd.0013037.s008.doc]

**Reply Letter**

**Editor comments:**

**Journal Requirements:**

***Comment 1:*** *Please ensure that the CRediT author contributions listed for every co-author are completed accurately and in full. At this stage, the following Authors/Authors require contributions: Li Ping Wong, Haridah Alias, Hai Yen Lee, Qinjian Zhao, Zhiwen Huang, Zhijian Hu, and Yulan Lin. Please ensure that the full contributions of each author are acknowledged in the "Add/Edit/Remove Authors" section of our submission form.
The list of CRediT author contributions may be found here: https://journals.plos.org/plosntds/s/authorship#loc-author-contributions*

**Reply:**

CRediT author contributions are now listed for every co-author.

***Comment 2:*** *Please provide an Author Summary. This should appear in your manuscript between the Abstract (if applicable) and the Introduction, and should be 150-200 words long. The aim should be to make your findings accessible to a wide audience that includes both scientists and non-scientists. Sample summaries can be found on our website under Submission Guidelines:
[https://journals.plos.org/plosntds/s/submission-guidelines#loc-parts-of-a-submission](https://journals.plos.org/plosntds/s/submission-guidelines#loc-parts-of-a-submission)*

**Reply:**

Added author summary/synopsis:

→ “The COVID-19 pandemic has shaped how people view vaccines, especially those approved quickly for emergencies. This study explores how these experiences affect trust in a potential mpox vaccine in China. A nationwide survey of 2,000 people found that about 68% were willing to get an mpox vaccine if one became available. Willingness was higher in less wealthy regions, like the Northeast and Northwest, compared to richer areas. Trust in emergency-approved vaccines, influenced by COVID-19 vaccination experiences, was the biggest factor driving people’s willingness to get vaccinated. Fear of mpox and taking preventive measures, like handwashing and avoiding close contact, also played a role. While many people said their trust in emergency vaccines increased after COVID-19, some still worried about how quickly these vaccines are approved and their long-term safety. The study suggests that clear communication, addressing concerns, and ensuring fair access to vaccines are key to building trust and acceptance for future health emergencies like mpox.”

***Comment 3:*** *Tables should not be uploaded as individual files. Please remove these files and include the Tables in your manuscript file as editable, cell-based objects. For more information about how to format tables, see our guidelines:*
[*https://journals.plos.org/plosntds/s/tables*](https://journals.plos.org/plosntds/s/tables)

**Reply:**

Tables are now removed from the system and inserted into the manuscript files.

***Comment 4:*** *Some material included in your submission may be copyrighted. According to PLOSu2019s copyright policy, authors who use figures or other material (e.g., graphics, clipart, maps) from another author or copyright holder must demonstrate or obtain permission to publish this material under the Creative Commons Attribution 4.0 International (CC BY 4.0) License used by PLOS journals. Please closely review the details of PLOSu2019s copyright requirements here: PLOS Licenses and Copyright. If you need to request permissions from a copyright holder, you may use PLOS's Copyright Content Permission form.
Please respond directly to this email and provide any known details concerning your material's license terms and permissions required for reuse, even if you have not yet obtained copyright permissions or are unsure of your material's copyright compatibility. Once you have responded and addressed all other outstanding technical requirements, you may resubmit your manuscript within Editorial Manager.
Potential Copyright Issues:*

*- Figure 1. Please provide a direct link to the base layer of the map (i.e., the country or region border shape) and ensure this is also included in the figure legend; and provide a link to the terms of use / license information for the base layer image or shapefile. We cannot publish proprietary or copyrighted maps (e.g. Google Maps, Mapquest) and the terms of use for your map base layer must be compatible with our CC BY 4.0 license.
Note: if you created the map in a software program like R or ArcGIS, please locate and indicate the source of the basemap shapefile onto which data has been plotted.
If your map was obtained from a copyrighted source please amend the figure so that the base map used is from an openly available source. Alternatively, please provide explicit written permission from the copyright holder granting you the right to publish the material under our CC BY 4.0 license.
If you are unsure whether you can use a map or not, please do reach out and we will be able to help you. The following websites are good examples of where you can source open access or public domain maps:
* U.S. Geological Survey (USGS) - All maps are in the public domain. (http://www.usgs.gov)
* PlaniGlobe - All maps are published under a Creative Commons license so please cite u201cPlaniGlobe, http://www.planiglobe.com, CC BY 2.0u201d in the image credit after the caption. (http://www.planiglobe.com/?lang=enl)
* Natural Earth - All maps are public domain. (http://www.naturalearthdata.com/about/terms-of-use/).*

**Reply:**

Following is the licensing rule from the our map resource mapchart.net:

“Licensing for maps:

This work is licensed under a Creative Commons Attribution-ShareAlike 4.0 International License. You are allowed to use, edit and modify any map (image) created with the Website or Mobile App for personal, non-commercial, or commercial use/publication freely, by adding any kind of reference to the Website or Mobile App in your work.

Some examples of an appropriate reference:

- Keeping the "Created with mapchart.net" or "Created with historicalmapchart.net" or "Created with MapChart" tag that each map has embedded.

- A simple link to the the front page or any other page of the Website or the Mobile App's store page.

- A text attribution on the page or inside the article/publication where the map is used (as a caption, in the credits, etc).”

**Revision:**

We have now added the following statement in the updated Figure 1:

“Source of base map: http://www.mapchart.net/china.html”

1. **INTRODUCTION/METHOD**

**Reviewer #1:**

***Comment 1:*** *Study authors note that they used convivence sampling, but fail to mention how this sample was generally recruited. How was the link to the survey sent to people, etc. that may bias this sample? Additionally information may help determine if the crude assumption of a design effect of 2 is adequate.*

**Reply:**

We clarify further and added in the manuscript.

→ Page 7, line 146-148 (line numbers refer to Tracked version of manuscript)

, the following statement is now added:

“A convenience sampling method was employed, with a survey company distributing the survey link through its existing database. Participants received the survey link via their mobile WeChat accounts.”

We added a reference citation noting the assumption of a design effect of 2.

→ Page 7, line 151-153, added:

“ The sample size was multiplied by the predicted design effect of two to account for the use of convenience sampling and an online survey [16]. Hence, the optimal sample size was set to 768 (384 x 2).”

***Comment 2:*** *How many provinces were included? Given the goal sample size of over 750 per province, the final sample size for the survey seems quite small. Additional information is required. I presume that this survey was powered to detect difference in response by province, though this was never explicitly stated. Then, there was no mention of province again following the beginning of the methods; this is concerning. Even by region, which perhaps contains provinces, there was not enough survey responses collected. This needs to be clarified.*

**Reply:**

Unfortunately, our survey only queried participants about the region they were from, using the following categories: [1] Northern, [2] Northeastern, [3] Eastern, [4] Southern Central, [5] Southwestern, [6] Northwestern. Given this regional categorization, our analysis was not powered to detect differences at the provincial level.

We add in the limitation, line 562-567:

→ “Furthermore, it is also important to note that the decision to categorize responses by region rather than by province limits the ability to analyze provincial-level variations, which may have important consequences. Given the diverse socio-economic, cultural, and healthcare landscapes across China's provinces, aggregating data at the regional level may conceal significant differences in vaccine confidence. This limitation affects the applicability of findings for policymakers, as province-specific insights are crucial for tailoring interventions.”

***Comment 3:*** *Given the lack of mention of human-to-human transmission in the paper’s introduction, what did study authors explicitly mean with respect to “avoiding close contact, practicing good hygiene, avoiding face-touching, using personal protective equipment (PPE), and staying informed” in their survey questionnaire? What was the precise language used and did it use language suggesting just animal-to-human or both animal-to-human and human-to-human transmission possibilities?*

**Reply:**

Added in line 126-129:

→“Specifically, we examine preventive behaviors such as avoiding close contact, practicing good hygiene, avoiding face-touching, using personal protective equipment (PPE), and staying informed by keeping updated with public health guidelines and advice from health authorities regarding mpox [14,15].”

As seen in our questionnaire, the Avoid Close Contact implies **“**Avoid close physical contact with people who have a rash or are suspected to be infected with Mpox.”

We have now rephrased the sentence in line 190-192:

→“To assess preventive behaviors against mpox, participants were asked five questions related to avoiding close contact.....” is now **rephrased** to:

“To assess preventive behaviors against mpox, participants were asked five questions related to avoiding close contact with people who have a rash or are suspected of being infected with mpox,.....”

***Comment 4:*** *The introduction mentions development of new vaccines, but fails to mention that an already existing JYNNEOS vaccine is available for high-risk persons. First, this should be mentioned somewhere in the paper as study authors leave out this possibility all together. Second, was this considered in the survey?*

**Reply:**

We acknowledge that JYNNEOS was not mentioned or considered in our survey. However, as per the suggestion, we have now incorporated the following clarification in lines 111–118:

→ “It is important to note that while existing vaccines used to protect against mpox provide some level of immunity, their limitations should be acknowledged. JYNNEOS, a non-replicating, live attenuated vaccine, is FDA-approved for the prevention of both smallpox and mpox [12]. However, JYNNEOS was originally developed as a smallpox vaccine with potential cross-protection against other orthopoxviruses rather than being explicitly designed for Mpox [13]. Therefore, the development of vaccines specifically designed for mpox, along with vaccine acceptance, remains a key priority in addressing its emergence as an infectious disease.”

***Comment 5:*** *Line 210-212: Please correct missing ethics statement.*

*There is no missing ethics statement we included i) A statement that formal consent was obtained (must state whether verbal/written) OR the reason consent was not obtained (e.g. anonymity).*

We have previously mentioned the ethics statement in line 148-150:

→“Writen consent to participate was obtained through a consent statement displayed on the first page of the online survey, where participants were required to click an agreement button to proceed. ”

Further we also enhance ethical statement by added the following statement:

Added line 147-1489:

→“Participants were informed about the voluntary nature of their involvement, and they could withdraw from the survey at any time without providing justification.”

Added line 153-157:

→“All data were handled confidentially and stored securely in compliance with data protection regulations. Participants did not receive any financial incentives for their participation in the study. There were no anticipated risks or potential harms associated with taking part in the research.”

***Comment 6:*** *What software was used for statistical analyses, and is the code publicly accessible?*

**Reply:**

Added line 241-242:

→“All statistical analyses were performed using the IBM SPSS Version 20.0 (IBM Corp, Armonk, NY, USA) [18].”

Regarding the code or syntax used for the analyses, we regret that it was not saved. However, to ensure transparency and reliability, we have made our data publicly available on Zenodo, accessible at the following link: <https://zenodo.org/records/14195496>.

***Comment 7:*** *Table 1 could contain much more comprehensive information, including key survey question results broken down by these demographic characteristics.*

**Reply:**

The main outcome of the survey is the willingness to be vaccinated with an EUA vaccine. As suggested, we have broken down this outcome by demographic characteristics. Other variables, such as knowledge, prevention practices, susceptibility, severity, fear, attitudes, and trust in EUA vaccines, serve as independent variables. However, due to the large number of these variables, we did not further stratify their findings by demographic characteristics.

Table 1 is now revised as suggested. More result statement are now also added in line 321-328:

→“Willingness to receive the mpox vaccine by demographics is shown in Table 1, with the highest willingness observed among those aged 26-35 (70.0%), though age and gender differences were not statistically significant. Education level played a key role (p<0.001), with higher willingness among those with a bachelor’s (75.0%) or postgraduate degree (70.1%) compared to lower education levels. Income also influenced willingness (p=0.030), with the highest acceptance among those earning >150,000 CNY (71.8%). Additionally, urban residents (75.3%) were significantly more willing than those from suburban (58.0%) and rural areas (58.1%) (p<0.001).”

**Reviewer #2:**

Overall, the study objectives are clearly articulated, and the nationwide cross-sectional design effectively provides a snapshot of mpox knowledge and willingness to receive the mpox vaccine.

***Comment 1:*** *However, it is not clear how the convenient sampling has been conducted. It is stated a company conducted random sampling across six regions in China. Could you elaborate on the targeted population and how participants were recruited for the survey? Specifically, how did they receive the survey link, and what methods were used to access the survey (e.g., email, social media, etc.)?*

**Reply:**

Thank you for your comment. Participants received the survey link via their mobile WeChat accounts. We have now provided more details.

Line 161-163, the following statement is now added:

→ “A convenience sampling method was employed, with a survey company distributing the survey link through its existing database. Participants received the survey link via their mobile WeChat accounts.”

***Comment 2:*** *In lines 457-458, you mention 'One notable limitation is the potential bias stemming from the non-random selection of participants in the online survey, which may not fully capture the diversity of the entire population.' However, this contradicts the methods section, where random selection is described. Please clarify this discrepancy for the reader.*

**Reply:**

We apologize for the inconsistency. We have revised the Methods section to remove any mention of random selection and have rephrased the sentence to ensure clarity and consistency.

Line 161-163, the following statement is now added:

→“A convenience sampling method was employed, with a survey company distributing the survey link through its existing database.”

***Comment 3:*** *In lines 135-137, you state, 'The sample size was multiplied by the predicted design effect of two to account for the use of convenience sampling and an online survey. Hence, the optimal sample size was set to 768 (384 x 2).' However, with six regions, the total optimal sample size should be 4608 (768 per region). The overall sample size was 2000. Does this discrepancy reflect a different sampling approach or is there another explanation? Additionally, could you specify how many surveys were excluded due to incompleteness?*

**Reply:**

We have acknowledged the study's limitation regarding the inability to achieve the optimal sample size, we rewrite the section in limitation. Regarding number of incompleteness, unfortunately, the survey did not record the number of surveys excluded due to incompleteness.

Line 548-561, the following limitation statement is now added:

→ “Of note, despite the intended target of 768 participants per region, actual sample sizes varied, with the Northwestern and Southwestern regions notably underrepresented (77 and 126 respondents, respectively). This uneven distribution may limit the generalizability of the findings, as smaller sample sizes reduce statistical power and increase the risk of bias in regional comparisons. Underrepresentation of certain areas may also skew results, as perspectives from these regions may not be fully captured, potentially affecting conclusions on vaccination willingness. Moreover, the imbalance in sample distribution may lead to a demographic profile that is disproportionately young, educated, urban, and higher-income. To improve representativeness and reliability, future studies should adopt targeted recruitment strategies and ensure a more balanced sample across all regions.”

***Comment 4:*** *Please include a statement regarding whether participants were compensated for their participation in the study, as part of the ethical approval section.*

**Reply:**

Added line 155-157:

→ “Participants did not receive any financial incentives for their participation in the study.”

**Reviewer #3:**

***Comment 1:*** *The methods section is well detailed with study objectives clearly explained. The section could use a little more detail on anticipated biases for the sampling method used. The sample size seems okay and the calculation is well detailed. Statistical analyses were ably explained.*

**Reply:**

Regarding anticipated biases for sampling method use, this is justified in limitation section line 542-543:

→“One notable limitation is the potential bias stemming from the non-random selection of participants in the online survey, which may not fully capture the diversity of the entire population.”

**Reviewer #4:**

***Comment 1:*** *Please clarify if the sampling method was convenience or randomized as the way the statement is written is confusing (the limitation section mentions that the sample is non-random).*

**Reply:**

We mistakenly stated random sampling, whereas this study actually employs convenience sampling.

Line161-163, the following statement is now added:

→“A convenience sampling method was employed, with a survey company distributing the survey link through its existing database. Participants received the survey link via their mobile WeChat accounts.”

***Comment 2:*** *The paragraph "Knowledge about mpox symptoms" (line 147) paragraph should be written in past tense.*

**Reply:**

We have now corrected the tense in the paragraph as suggested in line165-168:

→“The knowledge assessment **comprised** 8 items evaluating symptoms of mpox, including rash, fever, chills, swollen lymph nodes, fatigue, muscle aches, headaches, and respiratory symptoms. The response options **were** “true”, “false”, and “don't know”. A score of 1 **was** awarded for correct answers, while both “false” and “don't know” responses **received** a score of 0.”

***Comment 3:*** *Check for uniform capitalization for variables and options (eg. choose whether you want "Never" or "never" and keep it consistent throughout the methods)*

**Reply:**

We have corrected the tense in the paragraph as suggested.

***Comment 4:*** *Remove lines 241-243.*

**Reply:**

Based on our original submission, lines 241-243 state: “Chills were correctly noted by 41.9%, and swollen lymph nodes were recognized by 39.5% of respondents. The total knowledge score ranged from 0 to 8, with a mean of 3.6 (SD± 2.9).” We believe this information is important and should not be removed unless there was a different section being referred to. Please clarify if another part was intended.

***Comment 5:*** *Minor grammar errors that need to be fixed.*

**Reply:**

We have thoroughly reviewed the manuscript and made the necessary grammatical corrections, as reflected in the tracked changes.

2. **RESULTS**

   **Reviewer #1:**

***Comment 1:*** *Please see comments above; results may be inadequate if methods concerns are not addressed.*

**Reply:**

We have replied to each comments in the above section.

**Reviewer #2:**

***Comment 1:*** *The results section presents a comprehensive volume of data directly related to the research question. Additionally, it is beneficial that the characteristics of the respondents are clearly outlined at the beginning of this section. Overall, the results were conclusive and well-presented.*

**Reply:**

The characteristics of the respondents are outlined in lines 245–250. Furthermore, we have added more results descriptions.

Line 258-261, the following statement is now added:

→“Compared to participants in the undecided/somewhat unwilling/unwilling groups, those in the extremely willing/somewhat willing groups were more likely to have a higher education level, a higher annual family income, and reside in urban areas (all p<0.05).”

***Comment 2:*** *When mentioning CNY, please also include the equivalent amount in USD for clarity, especially for readers who may not be familiar with the currency exchange rates.*

**Reply:**

We have included a footnote of Table 1, noting that † 1 CNY = 0.137929 USD.

***Comment 3:*** *Figures/Tables: Please provide higher-resolution figures and tables to enhance readability (e.g. Appendix 2-5).*

**Reply:**

We have uploaded higher-resolution versions of the figures and tables (Appendix 2-5) to improve readability.

***Comment 4:*** *Please double-check Figure 2.*
- The value for preventive practice is stated as 0.066 in the text but is shown as 0.064 in the figure. Additionally, the values for knowledge and susceptibility do not match those presented in the text (lines 293-296).

**Reply:**

We have corrected the inconsistencies, line 332-333:

→The strongest positive association with willingness is trust in EUA vaccines (β= 0.441, p<0.001), followed by fear of the disease (β =0.096, p<0.001), and prevention practices (**β= 0.064, p=0.017**). Knowledge about mpox was also negatively associated with vaccination willingness; however, the association was not significant (β = -0.007, p = 0.715). Other significant but smaller positive effects include education (β =0.059, p=0.005) and susceptibility to the disease (**0.066, p=0.005**). ”.

***Comment 5:*** *Line 297: Is 'location' referring to 'birthplace'? This is unclear in Figure 2 and should be clarified.*

**Reply:**

We added birthplace location, line 335.

→“Negative associations were found with **birthplace** location (β =-0.053, p=0.012)...”

**Reviewer #3:**

***Comment 1:*** *The results section is detailed but text heavy. The two figures and one table at the end of the references section are not sufficient to provide enough visual presentation of the results.*

**Reply:**

We appreciate your feedback. There may be a misunderstanding, as in addition to the two figures and one table, we also included five appendices in our submission, which provide further visual representation of the results.

**List of Figures:**

- **Figure 1:** Proportion of willingness to receive the mpox vaccine by region
- **Figure 2:** PLS-SEM for factors associated with willingness to receive an mpox vaccine

**List of Appendices:**

- **Appendix 1:** Survey Questionnaire
- **Appendix 2:** Proportion of Correct Responses for Knowledge of Mpox Symptoms
- **Appendix 3:** Risk Perception Towards Mpox
- **Appendix 4:** Proportion of Responses for Mpox Prevention Practices
- **Appendix 5:** Proportion of Responses for Attitudes Towards EUA Vaccines

**Reviewer #4:**

***Comment 1:*** *Methods stated that ethnicity was captured but it is not in Table 1 of results or the first paragraph of results. Was there a reason as to why ethnicity was not mentioned? If not, please add that stat to the table and results section.*

**Reply:**

Thank you for your observation. We acknowledge the error in our methods section, ethnicity was not captured in our study.

Line 177, “ethinicity” is now removed:

→“Personal details collected include age, gender, ethinicity, highest educational level, monthly household income, locality, and current residing region. ” is now **revised to**

“Personal details collected include age, gender, highest educational level, monthly household income, locality, and current residing region. ”

***Comment 2:*** *Line 238, please re-word the sentence (minor grammar error)*

**Reply:**

We rephrase the sentence in Line 267:

→“Fever was identified correctly by 50.4% of respondents, followed closely by rashes at 49.8%. ” is now changed to:

“Fever was recognized as a symptom of mpox by 50.4% of respondents, while rashes were identified by 49.8%.”

***Comment 3:*** *Please contextualize the monthly income (high vs middle vs low/below poverty line) for researchers outside of the country.*

**Reply:**

Line 249-255, the following statement is now added:

→“Regarding annual family income, as of 2022, the average disposable income per capita in China was approximately CNY 36,883 annually [19]. Assuming an average household size of about 3 people (based on recent demographic data) [20], the average annual household income would be CNY 110,649. In our study, an approximately equal proportion of participants reported annual family incomes in the CNY100,001–150,000 range (32.9%) and the >150,000 CNY range (28.5%), reflecting a relatively balanced distribution of middle-to-upper-income households.”

***Comment 4:*** *The images in appendix are hard to read, please ensure that they are readable in the full publication.*

**Reply:**

We have uploaded higher-resolution versions of the figures and tables (Appendix 2-5) to improve readability.

***Comment 5:*** *For the "descriptive analysis of vaccine preference" (lines 313-314), the percentages add up to 85% and not 100%. Please revise or clarify of why that is the case.*

**Reply:**

We report the full results, bringing the total to 100%. Line 341-345:

→“Descriptive analysis of vaccine preference shows that a minority, 12.7% (n=254), prefer an mRNA vaccine, while 38.3% (n=767) favor a traditional conventional vaccine, and 34.0% (n=679) indicated they would accept either type of vaccine. Meanwhile, 6.4% **(n=128**) stated they did not want to receive any Mpox vaccine, and 8.6% (**n=172**) were unsure about the difference between mRNA and traditional vaccines.”

3. **CONCLUSIONS**:

   **Reviewer #1:**

***Comment 1:*** *Please see comments above; conclusions may be inadequate if methods concerns are not addressed.*

**Reply:**

We have replied to all comments.

**Reviewer #2:**

***Comment 1:*** *The conclusion is well-supported by the data and clearly connects to the research question. However, clarifying the factors behind regional variations in vaccine acceptance could strengthen it. Exploring whether regions with higher willingness for the mpox vaccine show similar trends with other vaccines or campaigns would be valuable, as would discussing strategies to increase trust in the target population.*

**Reply:**

Added line 467-470:

→ “Regional disparities in vaccine acceptance in China remain uncertain in the literature. In the context of COVID-19 vaccine, some studies have reported higher COVID-19 vaccine acceptance in the more developed eastern region [36,37], while others have found lower acceptance in the same region [38,39]. ”

***Comment 2:*** *The limitations are addressed, but providing more detail on the varying sample sizes across regions would improve transparency.*

**Reply:**

Added line 548-558:

→“Of note, despite the intended target of 768 participants per region, actual sample sizes varied, with the Northwestern and Southwestern regions notably underrepresented (77 and 126 respondents, respectively). This uneven distribution may limit the generalizability of the findings, as smaller sample sizes reduce statistical power and increase the risk of bias in regional comparisons. Underrepresentation of certain areas may also skew results, as perspectives from these regions may not be fully captured, potentially affecting conclusions on vaccination willingness.”

***Comment 3:*** *The authors effectively explain how the data advance understanding of vaccine acceptance. While the public health relevance is clear, including specific recommendations for improving vaccine confidence would enhance the practical implications of the study.*

**Reply:**

Added in line 568-583:

→“Based on the findings of this study, specific recommendations to enhance vaccine confidence and acceptance include implementing comprehensive educational campaigns to increase awareness of mpox symptoms and risks, particularly in regions with lower knowledge levels. Strengthening public health messaging, even in the absence of reported cases, can help sustain preventive practices and preparedness. Efforts should also focus on addressing concerns regarding EUA vaccines by transparently communicating their rigorous approval processes and safety measures. Tailored outreach strategies should be developed to address regional disparities in vaccine acceptance, particularly in economically developed areas where hesitancy may be higher. Additionally, affordability concerns must be considered to ensure equitable access to vaccines across diverse socio-economic groups. Given the strong preference for traditional vaccines, building confidence in mRNA and other new vaccine technologies requires emphasizing their rigorous safety evaluations, highlighting successful real-world applications, and ensuring clear, transparent communication about their benefits and potential risks.”

**Reviewer #3:**

***Comment 1:*** *Conclusions and limitations are valid and relevant at detailing pubic health relevance of the study.*

**Reply:**

Thank you for your positive feedback. We appreciate your recognition of the study's public health relevance and the validity of our conclusions and limitations.

**Reviewer #4:**

***Comment 1:*** *The discussion is a bit lacking. There are multiple instances of claims being made with no evidence (eg. line 339 - 340, 353-355, 367-369, etc), please add citations for any claims made.*

**Reply:**

The statements in lines 339-340, 353-355, and 367-369 are either not relevant for additional references or have already been cited. However, we wonder if there may have been an error in the line numbers provided. Nonetheless, we have thoroughly reviewed the manuscript and added references where applicable.

Added reference:

→Strengthening public health messaging, even in the absence of a local outbreak, could help sustain a higher level of precautionary measures and ensure preparedness should the situation change [22].

353-355 -already referenced

***Comment 2:*** *please fix some minor grammar errors*.

**Reply:**

We have thoroughly reviewed the manuscript and made the necessary grammatical corrections, as reflected in the tracked changes.

4. **SUMMARY AND GENERAL COMMENTS**:

   **Reviewer #1:**

***Comment 1:*** *Thank you for the opportunity to review this manuscript. Study authors present a survey on knowledge of mpox and acceptability of a possible future vaccine EUA. This topic is interesting and important as mpox continues to spread globally. However, I have significant concerns regarding the survey design and communication of such design.*

**Reply:**

We have addressed all comments regarding study design and made the releveant revisions.

**Reviewer #2:**

***Comment 1:*** *This study addresses a timely issue by examining post-COVID-19 confidence in EUA vaccines using a hypothetical mpox case. The manuscript is well-structured and offers valuable insights, but refining the methodology for clarity, specifically the process of convenience sampling and clarifying the sample size, would strengthen the conclusions. It's also important to note that this study was conducted in the absence of mpox cases and with limited media coverage in China. The limited media coverage and absence of mpox cases may have impacted participants' perceptions and vaccine willingness, potentially limiting the generalizability of the findings. These factors are appropriately acknowledged in the limitations and discussion sections.*

**Reply:**

Added line 580-5863, added the following statement:

→“Lastly, we acknowledge that this study was conducted during a period without reported mpox cases and with limited media coverage in China, which may have influenced public awareness, perceptions, and willingness to receive the mpox vaccine.”

***Comment 2:*** *Additionally, the term 'mpox' should be used consistently throughout the manuscript (e.g., in the title) to help reduce stigma. Overall, this is a relevant and well-conceived study that deserves publication.*

*Title: I would suggest using the term 'mpox' instead of 'monkeypox' to reduce stigma and avoid discrimination.*

**Reply:**

We appreciate your suggestion and have updated the title to use 'mpox' instead of 'monkeypox' .

→ Investigating post-COVID-19 Confidence in Emergency Use Authorization Vaccines: A Hypothetical Case of **Mpox**

***Comment 2:*** *Abstract:
- Conclusion: Consider adding strategies to strengthen public trust in vaccines within your target population.*

**Reply:**

Line 52-53, added the following statement:

→“This requires transparent communication about the rigorous approval process, continuous safety monitoring, and real-world effectiveness of EUA vaccines.”

***Comment 3:*** *Introduction:
- Line 119: Please define 'NTDs' upon first use to ensure clarity for all readers*

**Reply:**

We have defined "NTDs" as "neglected tropical diseases" upon first. Line 136.

***Comment 4:*** *Results:
- Line 227: 'A total of 2000 participants completed responses were received and included in the analysis.'
I suggest adding the number of surveys excluded due to incompleteness for clarity.*

**Reply :**

Thank you for your suggestion. Unfortunately, the number of incomplete responses was not captured by the survey company, limiting our ability to report the exact number of exclusions. However, all completed responses (N=2000) were included in the analysis.

***Comment 5:*** *Line 262-264: 'Many participants also worried about the effectiveness of EUA vaccines compared to fully approved ones (46.7% agreed, 29.5% strongly agreed) and potential unknown long-term side effects (48.8% agreed, 27.6% strongly agreed).'
This is an interesting finding. Is there an explanation for why the effectiveness of EUA vaccines is perceived as lower? This could be a valuable addition to the discussion section.*

**Reply:**

Added 392-399:
→“The belief that EUA vaccines are less effective than fully approved vaccines may arise from several factors. Firstly, EUA vaccines receive authorization based on interim clinical trial data rather than extensive long-term studies, raising concerns about their reliability [23,24[. Additionally, the EUA designation is often associated with an experimental or incomplete approval process. Past instances of expedited vaccine rollouts with unexpected side effects may further deepen skepticism [24,25]. Addressing these concerns through clear communication and transparency in vaccine development and authorization processes is crucial for maintaining public trust.”

***Comment 6:*** *Discussion:
- Line 349-352: 'The results on attitudes toward EUA vaccines revealed that many participants expressed concerns about the rapid approval process, quality control, and doubts about their effectiveness compared to fully approved vaccines.'
This is an interesting topic. Are there other publications that discuss why an EUA vaccine may be perceived as less effective?*

**Reply:**

Added reference line 396:
→ “Past instances of expedited vaccine rollouts with unexpected side effects may further deepen skepticism [24,25].”

***Comment 7:*** *Lines 365-366: I would suggest adding information about the guidelines and Standard Operating Procedures (SOPs) for EUA vaccines in China to provide more context.*

**Reply:**

Added line 437-446:

→ “In China, the National Medical Products Administration (NMPA) has established clear guidelines for EUA vaccines to ensure that safety and efficacy. The guidelines for vaccines development in China require comprehensive preclinical studies, including laboratory and animal testing, to evaluate the vaccine's safety and immunogenicity. While clinical trials typically progress through Phases I, II, and III to evaluate a vaccine’s safety and effectiveness, EUA allows for expedited approval based on interim data from early-phase trials, requiring at least Phase II data, if not Phase III. Even under accelerated timelines, these trials must meet stringent scientific and ethical standards. Vaccine manufacturers must adhere to good manufacturing practices to ensure the consistency and quality of vaccine production. Additionally, post-authorization monitoring is also a critical component, with robust pharmacovigilance systems in place to track adverse events and long-term effects after the vaccine is deployed [34]. ”

***Comment 8:*** *Lines 371-379: This is an interesting statement. What was the rationale behind China’s decision not to use mRNA vaccines for COVID-19? Is there more information on the reasoning behind this choice?*

**Reply:**

Added line 421-431:

→ “During the COVID-19 pandemic, China's decision to primarily use inactivated virus vaccines, instead of mRNA vaccines was influenced by a combination of scientific, logistical, and strategic factors. One key reason was the familiarity and established track record of inactivated vaccine technology, which had been used for decades in China and globally for diseases such as polio and Hepatitis A [29]. This established track record likely bolstered confidence in the safety and effectiveness of inactivated vaccines among regulators, manufacturers, and the general public Additionally, China prioritized domestic production capacity to ensure supply security and reduce dependence on foreign pharmaceutical companies [30]. Another factor was the challenges China faced in mRNA vaccine development during the early phase of the pandemic, including limited expertise and restricted access to specialized technologies. “

Added line 449-454:

→“Nevertheless, the country has since emphasized the importance of learning from the West’s advancements in mRNA vaccine technology and has called for global collaboration with an open-minded approach [35]. Therefore, there is a need to enhance public trust in mRNA vaccine technology for future pandemics as well as in the development of vaccines for NTDs such as mpox, as the future of vaccine development is increasingly shifting toward mRNA due to its superiority over traditional vaccines. ”

***Comment 9:*** *Lines 287-288 and 382-385: 'These findings indicate regional variation in vaccine acceptance, with higher rates in the northeastern and northwestern areas.'
Are there any previous studies or known factors that explain why these regions may have higher vaccine acceptance? Has this trend been observed in other vaccination campaigns or governmental initiatives in those regions?*

**Reply:**

Added line 467-470:

→“Regional disparities in vaccine acceptance in China remain uncertain in the literature. In the context of COVID-19 vaccine, some studies have reported higher COVID-19 vaccine acceptance in the more developed eastern region [36,37], while others have found lower acceptance in the same region [38,39].”

***Comment 10:*** *Lines 390-392: 'Tailored communication strategies and outreach efforts could be vital in boosting vaccine confidence, particularly in regions with low acceptance.'
What specific strategies could be implemented? Do similar measures already exist from other campaigns?*

**Reply:**

Added line 474-480:

→“Similar strategies have been successfully employed during the COVID-19 vaccination campaign, where digital platforms, community and healthcare workers engagement, and transparent communication were used to address hesitancy and improve coverage [40]. Additionally, while vaccination rates in Chinese cities remain relatively high due to smooth mobilization and strong health risk awareness, rural areas have faced challenges in uptake. To address this, targeted efforts such as free shuttle services, extended vaccination hours, mobile healthcare teams, and door-to-door education campaigns have been implemented to improve coverage [41].”

***Comment 11:*** *Lines 392-394: Please delete the duplicated sentence for clarity.*

**Reply:**

The duplicated sentence has been deleted.

***Comment 12:*** *Limitation:
- Line 464-466: 'For example, while the Eastern and Southern Central regions have relatively large sample sizes, other regions, such as the Northwestern and Southwestern, have significantly fewer participants.'
What factors contributed to this imbalance in participation?*

**Reply:**

Added line 550-554:

→“The imbalance in participation may be influenced by regional socioeconomic disparities in China. The Eastern and Southern Central regions, which have relatively large sample sizes, are more economically developed, urbanized, and have higher levels of education and digital engagement. These factors likely contribute to greater awareness of health-related issues and a higher willingness to participate in online surveys. ”

**Reviewer #3:**

This is great write up on the topic, worth acceptance pending minor revisions.

**Reply:**

Many thanks for your valuable comments.

**Reviewer #4:**

Thank you, it was a pleasure to review your publication. This manuscript is well done and thought out; it just needs a few edits and some points of clarification. I think this will add to the body of literature that aids to fight misinformation and fear surrounding vaccines on a global level. While the study had a simple, cross-sectional design, the analyses were well done. Furthermore, the study reported crucial data regarding attitudes, knowledge, and risk perception surrounding Mpox and vaccines in general. I recommend this manuscript for "Minor Revision."

**Reply:**

Many thanks for your valuable comments.

5. **REPRODUCIBILITY**

   To enhance the reproducibility of your results, we recommend that authors of applicable studies deposit laboratory protocols in protocols.io, where a protocol can be assigned its own identifier (DOI) such that it can be cited independently in the future. Additionally, PLOS ONE offers an option to publish peer-reviewed clinical study protocols. Read more information on sharing protocols at <https://plos.org/protocols?utm_medium=editorial-email&utm_source=authorletters&utm_campaign=protocols>

**Reply:**

Our study does not involve a laboratory protocol, therefore, depositing a study protocol is not applicable. However, we have made our data publicly available on Zenodo, which can be accessed at the following link: <https://zenodo.org/records/14195496>. We have also now added this link in the revised manuscript.
